# The microscopic origin of the extreme glass-forming ability of Albite and B_2_O_3_

**DOI:** 10.1038/srep43022

**Published:** 2017-02-27

**Authors:** Edgar D. Zanotto, Daniel R. Cassar

**Affiliations:** 1 Department of Materials Engineering, Center for Research, Technology and Education in Vitreous Materials, Federal University of São Carlos, São Carlos, SP, Brazil

## Abstract

Understanding the conditions that favour crystallisation and vitrification has been a longstanding scientific endeavour. Here we demonstrate that the extremely high glass-forming ability of unseeded supercooled Na_2_O·Al_2_O_3_·6SiO_2_ (Albite) and B_2_O_3_—known for decades as “crystallisation anomaly”—is caused by insufficient crystal nucleation. The predicted temperatures of the maximum homogeneous nucleation rates are located well below their glass transition temperatures (T_g_), in a region of very high viscosity, which leads to extremely long nucleation time-lags and low nucleation rates. This behaviour is due to the remarkably small supercoolings where the glass transition occurs for these liquids, which correspond to a very small driving force for crystallisation at and above the T_g_, where crystallisation is normally observed. This meagre nucleation ability is caused by the significant difference in the structures of the supercooled liquids and their isochemical crystals. These findings elucidate the cause behind the crystallisation anomaly, and could be used for the design of other oxide glasses that are extremely stable against crystallisation.

The most stable thermodynamic state of matter below the melting point or *liquidus* is the crystalline state, where the atoms are well organized in a periodic lattice at short-, intermediate-, and long-range distances. The vast majority of natural materials on Earth’s crust are crystalline. To make glasses one has to deceive the natural solidification path: crystallisation^1^. There has been a long-standing quest to understand the conditions that favour crystallisation and vitrification of supercoooled liquids (and the nature of the vitreous state) ever since glasses became a major topic within condensed matter physics and materials science and engineering.

Most ionic, covalent, van der Waals, mixed bonded, metallic, and inorganic liquids crystallise within laboratory time scales when they are cooled down somewhat below their respective equilibrium melting points or *liquidus* temperatures (*T*_*m*_). Turning to the realm of inorganic glasses, researchers and engineers frequently struggle against unwanted spontaneous crystallisation (also known as *devitrification*) when they try to make glasses in their laboratories or industries; whereas others try to design and control crystallisation in glass article interiors to produce a type of nanoporcelain known as *glass-ceramics*^2^,^3^,^4^. In fact, “crystallisation” is so important that it is the most frequent keyword used by researchers in the last 200 years of glass science history^5^.

However, to the best of our knowledge and to our great surprise, crystallisation has never been reported to occur in pure unseeded supercooled Na_2_O·Al_2_O_3_·6SiO_2_ (Albite) and B_2_O_3_ liquids, at atmospheric pressure. These materials display outstanding glass-forming ability (when cooled from the liquid state), and are likely the most stable stoichiometric oxides against crystallisation (on heating from the glassy state) discovered to-date! In the glass science field, a “stoichiometric glass” is one that has a thermodynamically stable isochemical crystalline phase. This definition should not be confused with the traditional “stoichiometry”, that is, the calculation of relative quantities of reactants and products in chemical reactions.

In this article we will focus on both glasses: in Albite glass because it is also outstandingly chemically durable and may, for instance, provide an excellent matrix for the immobilization of radioactive fission products; and on B_2_O_3_ because this is one of the main oxide glass-formers used in a plethora of commercial glass products. In addition, Albite is a strong liquid, whereas B_2_O_3_ is fragile, and enough information on their structures and properties is available to perform sufficient calculations to develop a strong argument to explain their outstanding glass-forming ability. A liquid may be strong or fragile depending on the value of its fragility index m, defined by m = *d*log(η(T))/*d*(T_g_/T)|_T = Tg_, where η is the equilibrium viscosity and T_g_ is the glass transition temperature. Note that this fragility index is not related to the mechanical properties of the material.

As an example of the types of experimental tests performed with these glasses, in the fifties Schairer and Bowen^6^ showed how difficult it is to crystallise Albite from its own melt; one sample was held at 1025 °C (close to the temperature of the predicted maximum growth rate) for five years without crystallising^7^. Uhlmann^8^ coined the term crystallisation “anomaly” when trying to decipher this unusual behaviour, which that is also shown by B_2_O_3_ glass, which only crystallises after seeding and under very high (GPa) pressures^9^. Albite has been shown to crystallise at atmospheric pressure only when Albite crystal seeds are introduced^10^,^11^,^12^. In a very recent article, Siqueira *et al*.^13^ demonstrated that even a (carbon-free) gel-derived Albite glass—and gels easily devitrify—did not crystallise when heated at the temperature of maximum growth rate. One of us (EDZ) has tried to crystallise an unseeded B_2_O_3_ glass with different thermal treatments without success.

These experiments indicate that the main challenge preventing crystallisation is in the formation (nucleation) of a crystalline phase (Albite and B_2_O_3_), and perhaps even in its growth (B_2_O_3_). This crystallisation anomaly has been a matter of debate for many decades!

The typical stoichiometric oxide glass forming compositions (such as Albite and B_2_O_3_) normally crystallise in laboratory time scales when heated at any temperature somewhat below their melting points and above their T_g_, and also during non-isothermal DSC (differential scanning calorimetry) runs. However, crystallisation does not occur in Albite and B_2_O_3_ within the time scales of DSC experiments.

Therefore, understanding the reasons why these two glass-formers display this abnormal reluctance to crystallization in heating experiments and outstanding glass-forming ability (in the cooling path from the molten state) is very relevant from the physics, chemistry, and materials science point of view. From a broader scientific perspective, this study could also provide further insight into the nature of the vitreous state and be used for the design of very stable glasses.

In this article we dwell on and calculate three kinetic properties of supercooled liquids that control crystal nucleation and crystal growth: the steady-state homogeneous crystal nucleation rates, I_st_(T); the nucleation time-lags, τ(T); and the crystal growth rates, U(T), for Albite and B_2_O_3_. Because the homogeneous nucleation kinetics are too slow to be measured in these liquids, we rely on estimated values of I_st_(T) and τ(T). Finally, we use literature data to compare the similarities and differences between the atomic structures of the parent liquids and their isochemical crystal phases. We sought for any thermodynamic, kinetic, or structural signs that could shed light on the lack of observable crystallisation in the supercooled Albite and B_2_O_3_ liquids.

## Thermodynamic and Kinetic Parameters that Control Crystal Nucleation and Growth

Reluctant crystallisation could be due to either exceptionally low steady-state crystal nucleation rates, very long induction periods for nucleation, and/or extremely low crystal growth rates. We will now discuss the governing equations for these three kinetic properties, and at their main controlling parameters, that can be calculated or measured, which are: the crystallisation driving force, the nucleus-melt interfacial energy, and the effective diffusion coefficient. We will then start with a brief definition of these parameters, before discussing the crystal nucleation and crystal growth models.

### Driving Force for Crystallisation (Δμ)

Supercooled liquids always have a higher Gibbs free energy (*G*_*l*_) than their stable isochemical crystal phases (*G*_*c*_). The driving force for crystallisation is defined as Δμ ≡ −ΔG = *G*_*l*_ − *G*_*c*_ and is positive for temperatures below the melting point^14^,^15^. In addition, the absolute value of Δμ increases with decreasing temperature from the melting point (that is, increasing undercooling). Equation (1) provides a way to calculate Δμ for a closed system under isobaric condition^14^,^15^.


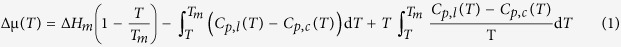


In the above equation, Δ*H*_*m*_ is the molar enthalpy of melting, *C*_*p,l*_ is the molar heat capacity of the supercooled liquid, *C*_*p,c*_ is the molar heat capacity of the crystal, and *T* is the absolute temperature. When the driving force for crystallisation is expressed in units of volume rather than in moles of substance, the conversion requires the knowledge of the molar volume, V_m_:


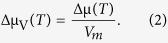


**Figure 1:**
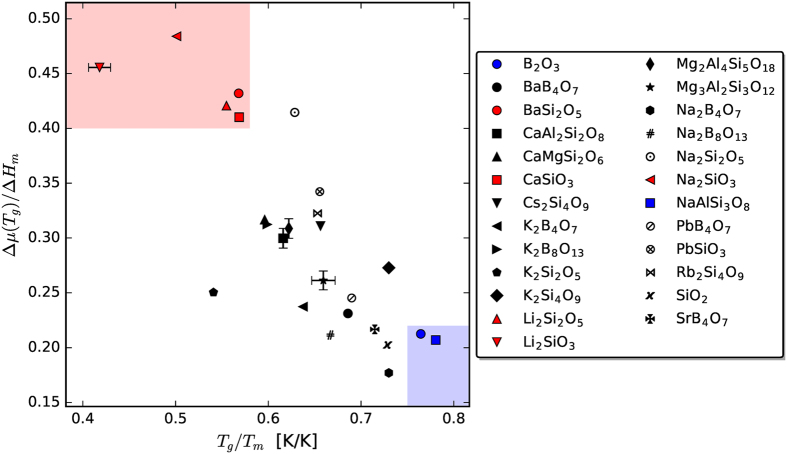
Reduced driving force for crystallisation evaluated at T_g_ *versus* reduced T_g_ for a number of oxide glass-formers. The red points refer to compositions that reveal internal homogeneous crystal nucleation, whereas the blue points refer to the two known stoichiometric oxide liquids that do not show any signs of nucleation (B_2_O_3_ and Albite). The remaining points refer to supercooled liquids that only reveal surface (heterogeneous) crystal nucleation. When available, the typical uncertainties are shown for some compositions. Thermophysical data were obtained from various sources^46^,^47^,^63^,^64^,^65^,^66^,^67^,^68^,^69^,^70^,^71^,^72^,^73^,^74^,^75^,^76^. Values of T_g_ were obtained by fitting viscosity data collected from the SciGlass database^77^.

Figure 1 shows a normalized plot of Δμ(T_g_)/ΔH_m_ versus T_g_/T_m_ (calculated and proposed here) for a series of glass-forming oxides, where three regions can be observed: the red area depicts the region and oxide glasses for which internal homogeneous crystal nucleation is readily observed; the region of the two glasses for which no crystallisation is observed is blue. All the other points refer to oxide glasses that only crystallise heterogeneously starting on their external surfaces. This plot is in agreement with and reinforces Turnbull’s^16^ observation that liquids with T_g_/T_m_ > 2/3 can be easily undercooled to the glassy state if they are free of crystalline seeds.

### Nucleus-Liquid Interfacial Energy (*σ*_*cm*_)

Unfortunately the nucleus-melt interfacial energy, *σ*_cm_, cannot be directly measured. However, it can be estimated using the Skapski–Turnbull^17^,^18^,^19^ expression:


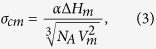


where *α* is a non-dimensional constant and N_A_ is Avogadro’s number. From both theoretical arguments and the fitting of the Classical Nucleation Theory equation to experimental crystal nucleation rates in oxide glass-formers that show measurable homogeneous nucleation, *α* is expected to be in the range of 0.4–0.6^20^. For glasses that do not display homogeneous nucleation *α* may be even higher.

### Viscosity and Diffusivity

The effective diffusion coefficient controlling viscous flow and supposedly crystal nucleation and growth in supercooled liquids can be calculated by the Eyring^21^ equation:


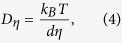


where *D*_*η*_ is the diffusion coefficient, *k*_*B*_ is Boltzmann’s constant, *d* is the jump distance, which is equivalent to the diameter of the diffusing molecules, and *η* is the viscosity. As *d* varies significantly less with temperature than *η*, for practical purposes it can be considered a constant equal to *d*_*o*_ ∼ (V_m_/N_A_)^1/3^.

Several viscosity equations are available in the literature^22^,^23^,^24^,^25^. In this work we considered the VFT^26^,^27^,^28^ which corresponds to Eq. (5). This equation together with Eq. (6) (also known as CWAM^29^,^30^) fit the viscosity data of oxide liquids between the T_g_ and T_m_ very well, but yield upper and lower bounds, respectively, for the extrapolated values of the equilibrium viscosity below T_g_. They are both used in this article.


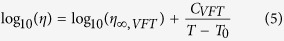



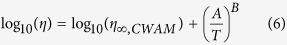


**Figure 2:**
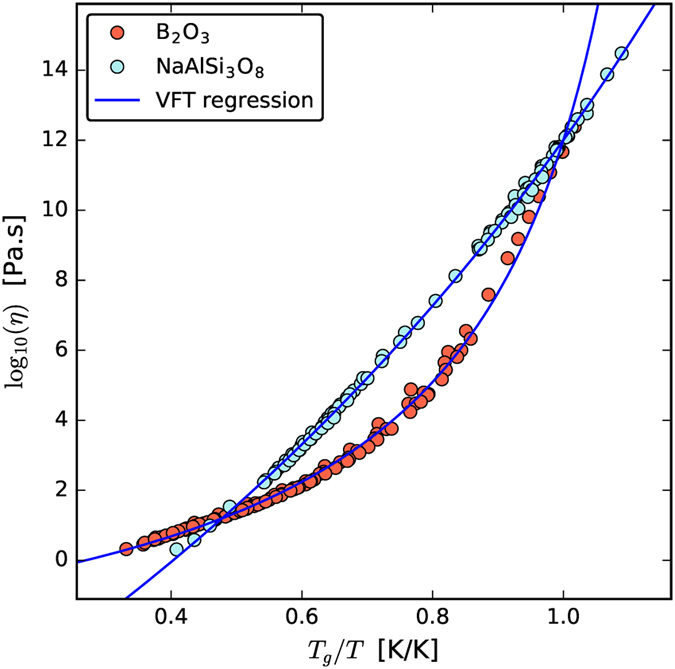
Viscosity data for Albite^78^,^79^,^80^,^81^,^82^,^83^,^84^,^85^,^86^ and B_2_O_3_^87^,^88^,^89^,^90^ and fitted curves using the VFT equation. These are examples of “strong”, almost linear (Albite) and “fragile”, highly curved (B_2_O_3_) liquids.

In these equations, *η*_*∞*_ is the asymptotic value of the equilibrium viscosity when *T *→* ∞*, and A, B, C, and T_0_ are adjustable parameters. Figure 2 shows viscosity data and fitted curves for Albite and B_2_O_3_ in an Oldekop–Laughlin–Uhlmann–Angell^31^,^32^,^33^ (OLUA) plot. The values for the adjustable parameters in this figure are shown in Table 1.

The actual diffusion coefficients controlling crystal nucleation and growth, *D*_*I*_ and *D*_*U*_, respectively, are unknown for oxide glass formers. Hence they have been largely calculated by the Eyring or Stokes-Einstein equations, *D*_*η*_^34^,^35^,^36^,^37^,^38^,^39^, which yield good results for various oxide glass formers^38^ at relatively high temperatures not far below the *liquidus. A*ccurate crystal growth studies revealed that this equation breaks down at deep supercoolings, at *T*_*d*_ ∼ 1.1–1.2*T*_*g*_^38^,^39^. Equilibrium viscosity and crystal growth kinetics are thus “coupled” from the melting point down to *T*_*d*_, but they diverge significantly below *T*_*d*_. For this reason, *T*_*d*_ is often denominated decoupling (or breakdown) temperature. However, to the best of our knowledge, signs of a similar breakdown of the crystal nucleation kinetics have not been clearly shown to-date. Therefore, in this article, we use the Eyring equation to approximate the diffusion coefficient controlling nucleation, and thus neglect the effects of a (possible) breakdown. In addition, as the crystal growth calculations are carried out above *T*_*d*_, as we will show later, there is no need to consider a (possible) breakdown. Finally, at least Albite is a very “strong” liquid for which the breakdown is much less likely.

## Theories and Models of Crystallisation and their Applicability

Crystallisation may be divided into the following two processes: the formation of stable crystalline nuclei, and their volume change with time and temperature. The first is termed crystal nucleation and the second is termed crystal growth. In the following subsections we will briefly introduce the main models for these two processes.

### Classical Nucleation Theory

The Classical Nucleation Theory (CNT) provides the physical description of the crystal nucleation process. Here we introduce the basic equations for the steady-state homogeneous crystal nucleation rate, *I*_st_,


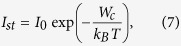



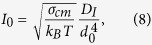


and the time-lag for crystal nucleation, τ (time necessary for the establishment of a stationary embryo size distribution up to the critical size),


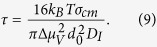


For spherical nuclei, the work of the formation of a nucleus with a critical size, often denoted as the thermodynamic barrier for crystal nucleation, *W*_c_ is given by Eq. (10).


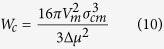


A nucleus is called “critical” when its growth is spontaneous resulting in a decrease of the system’s Gibbs free energy. These relations will be used in the present study to estimate the crystal nucleation rates in the framework of CNT. In the above equations, *σ*_*cm*_ is the crystal-liquid interfacial energy of the critical nucleus (in units of J/m^2^, assumed to be the same used in the macroscopic growth equations); *k*_B_ is the Boltzmann constant; *T* is the absolute temperature; Δμ_v_ is the driving force for crystallisation in units of J/m^3^; and *D*_*I*_ is the effective diffusion coefficient determining the processes of aggregation of “structural units” to the crystalline clusters, estimated here by the Eyring expression (Eq. 4). Hence, the expression for the nucleation rate becomes


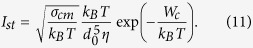


The actual magnitudes of I_st_ cannot be calculated with a reasonable degree of accuracy due to the uncertainty in *σ*_cm_^40^,^41^ and its cubic exponent inside an exponential. However, the temperature dependence of I_st_ and the location of its maximum, T_max_, can be adequately calculated using properly estimated values of *σ*_cm_, as we will do in this article.

Certain defect sites and solid impurities can reduce the thermodynamic barrier and the time-lag for nucleation. The formulation presented above can be maintained with correction factors^42^ given by Eqs (12) to (15).


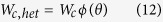



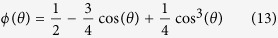



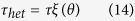



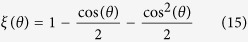


In the above equations, W_c,het_ is the thermodynamic barrier for heterogeneous crystal nucleation, ϕ and ξ are functions that depend on the contact angle between the nucleus and the substrate, θ, and τ_het_ is the time-lag for heterogeneous nucleation.

A key parameter that can be measured for glass-forming systems is the nucleation time-lag, τ, as a function of temperature, as shown in ref. 43. For all oxide glass-forming liquids tested to-date, these nucleation time-lags only become detectable (reach laboratory time scales) in the neighbourhood of their respective T_g_ and keep increasing upon further supercooling.

**Figure 3:**
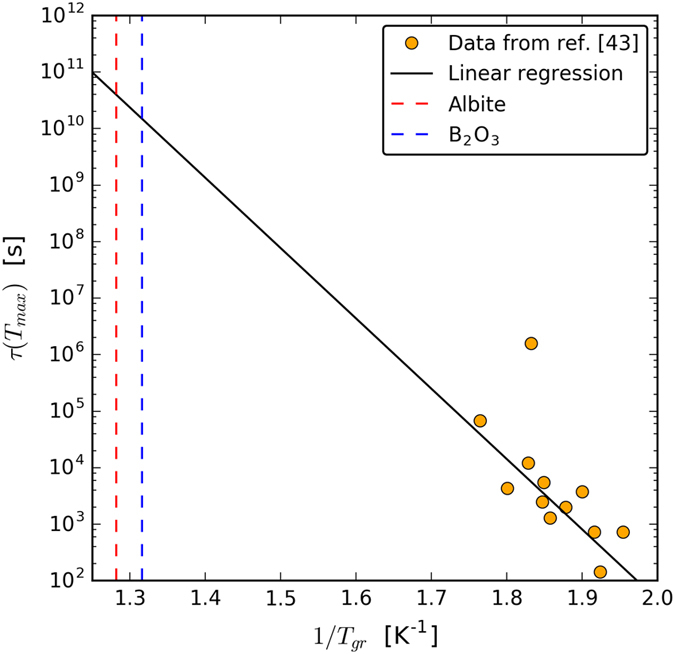
Experimental values of the nucleation time-lags at T_max_ for 12 oxide compounds that show homogeneous nucleation in laboratory time scales. Data from ref. 43. The dashed vertical lines refer to the expected values for B_2_O_3_ and Albite from a linear extrapolation.

Figure 3 shows the experimental values of τ at the temperatures of maximum crystal nucleation rate, T_max_. These are data for 12 substances that show homogeneous nucleation in laboratory time scales. It is quite clear that τ increases dramatically (please note the logarithm scale) with increasing values of the reduced glass transition temperature T_gr_ = T_g_/T_m_. An extrapolation of this plot for the cases of Albite (T_gr_ = 0.78) and B_2_O_3_ (T_gr_ = 0.76) gives an estimated time-lag of more than 10^10^ seconds (about 300 years).

**Figure 4:**
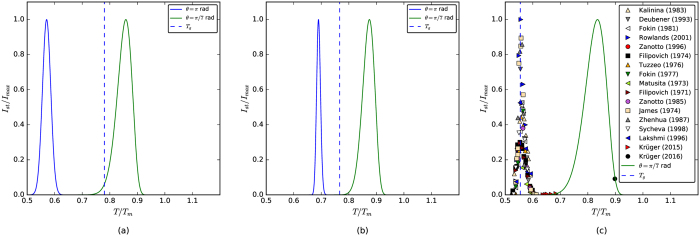
Normalized crystal nucleation rate curves *versus* reduced temperature for the cases of homogeneous nucleation (θ = π rad) and heterogeneous nucleation (θ = π/7 rad) for (**a**) Albite and (**b**) B_2_O_3_. A plot for lithium disilicate is shown in (**c**) to compare our calculations for Albite and B_2_O_3_ with experimental homogeneous^91^,^92^,^93^,^94^,^95^,^96^,^97^,^98^,^99^,^100^,^101^,^102^,^103^,^104^ and heterogeneous^44^,^45^ crystal nucleation data. The value of I_max_ for the heterogeneous crystal nucleation data was taken from Fig. 6 of ref. 45. The vertical dashed line is the estimated T_g_ (where the viscosity is 10^12^ Pa.s.).

Figure 4(a) and (b) shows the cupola shape of the crystal nucleation rate curves for glassy Albite and B_2_O_3_. The homogeneous nucleation (θ = π rad, blue line) rate maxima are located well below the glass transition temperatures, which suggest that the crystal nucleation rates at and above T_g_ (where the kinetic phenomena start to be measurable for most glass-forming systems) could be extremely small.

Compare these figures with Fig. 4(c), which shows experimental data and calculations for lithium disilicate (LS2), a supercooled liquid that undergoes internal homogeneous nucleation. The position of the *homogeneous* crystal nucleation peak for LS2 in the respective T/T_m_ scale is not much different from the same peak for Albite, but the value of T_g_/T_m_ for LS2 is equal to its homogeneous crystal nucleation maximum. We will discuss this issue in more detail below.

Experimental data for heterogeneous crystal nucleation of LS2^44^,^45^ in a supercooled liquid contained in a Pt pan is well described using a value of θ = π/7 rad. When we used this value of θ in the calculations for Albite and B_2_O_3_ an interesting picture emerged: the *heterogeneous* nucleation peak is in fact well above the glass transition temperature for these two compositions, a temperature region where the expected nucleation time-lags are much shorter. This means that heterogeneous crystal nucleation may be possible in these materials with a powerful nucleation agent (such as seeding with their own isochemical crystals).

To calculate the nucleation curves of Fig. 4(a) and (b), we used Eq. (11) with d_0_ calculated from the molar volume, as shown in Subsection 2.3. In this context we calculated σ_cm_ via Eq. (3) with α = 0.5 and Δμ_v_ by Eqs (1) and (2). Table 2 shows all the physical and thermodynamic parameters used in the calculations. The heat capacity for the crystalline phase is shown in Eq. (16) for Albite^46^ and Eq. (17) for B_2_O_3_^47^:


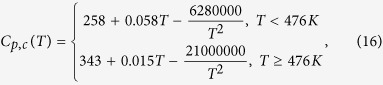



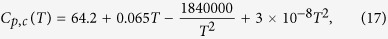


with C_p,c_ in J/K.mol and T in Kelvin.

The values of equilibrium viscosity (rather than the non-equilibrium viscosity), given by Eq. (5) were used even below the T_g_ of each glass because the structural relaxation times, τ_rel_, are significantly shorter than the crystal nucleation time-lags, which is similar to the average time of formation of the first critical nucleus, <τ>. In fact, the temperature where τ_rel_ = <τ> is the so-called supercooled liquid metastability limit (SCLML) or kinetic spinodal, T_KS_^48^,^49^,^50^. Our estimates of T_KS_ for three oxide liquids (to be published) demonstrate that T_KS_/T_m_ < 0.50, whereas T_max_/T_m_ for Albite is 0.55 and is 0.65 for B_2_O_3_. Therefore, these two glasses should relax to the supercooled liquid state before the first critical nucleus is formed. Hence, the use of equilibrium viscosity to calculate nucleation kinetics above T_KS_ is a very reasonable assumption. However, in any case, if instead the non-equilibrium viscosity were used in the calculations, the resulting T_max_ would be located at even deeper supercoolings. In that case, the conclusion so far reached (long nucleation time-lags) would be reinforced. We have also used Eq. (6) for the equilibrium viscosity in the above discussed calculations and the results did not significantly change.

**Figure 5:**
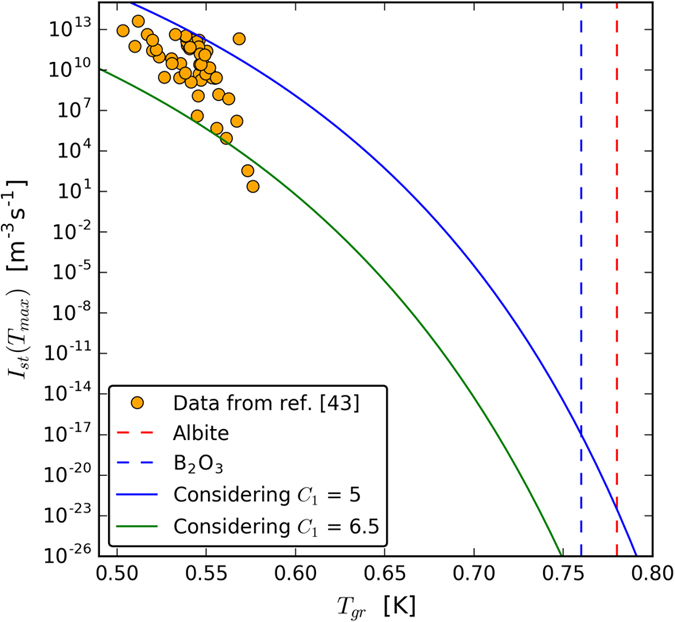
Maximum experimental steady-state homogeneous crystal nucleation rates at T_max_ *versus* reduced T_g_ (T_gr_) for 51 oxide glass-formers that undergo internal homogeneous nucleation. Data from ref. 43. The full line is the predicted I_st_(T_max_) using Eqs (3), (9), (16) and (17) from Gupta *et al*.^105^ In these calculations we considered reasonable values of C_2_ ≡ C_VFT_/T_m_ = 4, I_0_ = 10^42^ m^−3^ s^−1^, and C_1_ ≡ (Δμ/ΔH_m_)^2^W_c_/k_B_T_m_ equal to 5.0 and 6.5. The vertical dashed blue line refers to the T_gr_ of B_2_O_3_ and the red line to Albite.

A key element for the rationale of the present article is that the maximum experimental steady-state homogenous crystal nucleation rates, I_st_(T_max_) drastically decrease with increasing T_gr_ of the parent glass. The overall picture is shown in Fig. 5 and has also been corroborated by theoretical calculations as discussed thoroughly by Fokin *et al*.^43^. This figure indicates that the homogeneous nucleation rates in Albite (T_gr_ ∼ 0.78) and B_2_O_3_ (T_gr_ ∼ 0.76) are likely extremely small.

Apart from three outliers, the theoretical prediction is sufficient to describe all the other experimental data within the uncertainty (that is, the range considering C_1_ from 5.0 to 6.5.) The data scatter makes it difficult to affirm that the temperature dependence of the theoretical prediction is different from the experimental data. Perhaps the experimental data show a steeper decrease than the theoretical prediction, but if this is the case, our conclusions would not be affected.

#### Crystal Growth Models

Experimental crystal growth rates in oxide glass-formers are reasonably well described by one of the following three classical models: *normal* or continuous growth, *screw dislocation* growth, and 2D *secondary nucleation growth*^20^. The first two growth modes are quite common for oxide glass-formers and share the same expression:


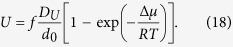


The difference between these two growth models is on the fraction of sites on the growing crystal interface available for atomic attachment, *f*. For materials with a small melting entropy^51^, ΔH_m_/T_m_ < 2R (R is the gas constant), *f* is independent of the temperature and approximately 1. In such case, *normal* growth prevails. Otherwise, if 0 < *f* < 1 and is temperature dependent, then the *screw dislocation* mechanism governs crystal growth. The parameter *f* can be estimated using Eq. (19) if one considers screw dislocations that form Archimedean spirals^52^ at the advancing liquid–crystal interface.


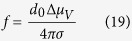


In the above equations, *U* is the crystal growth rate, *D*_*U*_ is the effective diffusion coefficient controlling crystal growth, and σ is the crystal-liquid surface energy (normally assumed to be size and temperature independent and equal to σ_cm_). When *f* = 0 (perfect crystals) then crystal growth must occur via 2D secondary nucleation, each crystal layer being formed by a secondary nucleus on the top of the primary nucleus and so on. The expression for this mechanism is well documented in Gutzow & Schmelzer’s book^20^.

**Figure 6:**
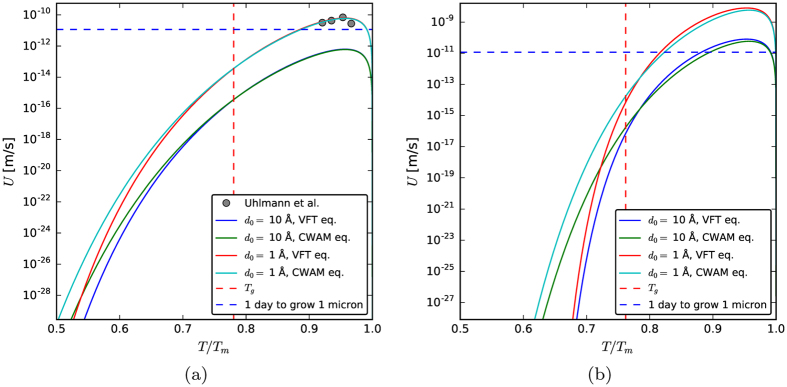
Simulations with the screw dislocation crystal growth model for (**a**) Albite and (**b**) B_2_O_3_. The experimental data points in (**a**), referring to the growth of crystalline layers in seeded Albite glass observed by Uhlmann *et al*.^10^, match the curve calculated with *d*_*o*_ = 1 Å. The horizontal dashed line refers to the value of growth rate for which the crystals would grow 1 μm in 1 day.

To calculate the crystal growth rates curves shown in Fig. 6, we used Eqs (18) and (19) and the same value for σ used in the Subsection 3.1 and two values for d_0_, of the order of magnitude of a chemical bond length. The experimental data points refer to the growth of crystalline layers observed by Uhlmann *et al*.^10^ after dusting an Albite glass surface with crystalline Albite mineral crystals.

Therefore to grow a crystal of 1 micron (that is resolvable under an optical microscope) one would need many years at the temperatures of maximum homogeneous nucleation, T_max_ ∼ 0.55T_m_ (Albite) and  ∼ 0.65T_m_ for B_2_O_3_. However, for temperatures well above T_g_, measurable growth occurs in a timeframe of hours, as shown in Fig. 6(a), where the four data points of Uhlmann *et al*.^10^ (obtained with seeded Albite glass) fit quite well the calculated curve with *d*_*o*_ = 1 Å. In summary, the maximum crystal growth rates are slow (U_max_ ∼ 1 Å/s for Albite and  ∼ 50 Å/s for B_2_O_3_), but growth can and has been observed above T_g_ and is not the problem hindering the crystallisation in supercooled Albite liquid, as shown by the experiments with seeded samples^10^,^11^,^12^. For B_2_O_3_ the theoretically estimated growth rates (by the screw dislocation mechanism) are even higher than those of Albite. However, we cannot draw firm conclusions about its growth kinetics because, to our knowledge, no published growth data are available for growth of B_2_O_3_ crystals at ambient pressure. One could also argue the growth rates in this glass could be described by another growth mechanism.

In any case, the exceptional resistance to crystallisation of both liquids clearly resides in their poor crystal nucleation kinetics, which was theoretically analyzed in the preceding sessions. In the following session, we dwell on the structures of the glass and their isochemical crystal phases in a quest for a structural reason for this poor nucleation ability.

## Structural Aspects of the Glasses and their Isochemical Crystals

It is known empirically^53^ that the densities of the parent glass and corresponding crystal phase are quite similar (Δρ/ρ_glass_ < 8%) for all stoichiometric oxide glasses that display internal homogeneous crystal nucleation in laboratory time/size scales. However, several stoichiometric oxide glasses that only display surface (heterogeneous) nucleation, or no nucleation at all, show higher values of Δρ/ρ_glass_. In the case of Albite, Δρ/ρ_glass_ = 15% and for B_2_O_3_ the density mismatch is approximately 34%.

The Δρ/ρ_glass_ ratio gives clear signs of structural similarity or dissimilarity between the parent glass and its isochemical crystal, hence Albite and B_2_O_3_ seem to have a significantly different structure from their crystalline phases. Let us consider two extreme cases: if the structure of the liquid is very close to that of its crystal form, then their densities must be similar and the necessary structural reorganization for crystallisation is facilitated by the reduction in the kinetic barrier for nucleation and reduction of the surface energy, but the thermodynamic driving force, Δμ_V_, will be weak. However, as the crystal nucleation rates exponentially diminish with an increase in σ_cm_^3^/Δμ_V_^2^ (Eq. 10), the role of σ_cm_ is stronger and prevails. Structural similarity (lower σ_cm_) is thus favourable for higher nucleation rates. The cases of Albite and B_2_O_3_, for which the densities (and inferentially the structures) of the liquids strongly differ from those of the isochemical crystals, correspond to a high surface energy, which lead to smaller nucleation rates. As the values of α (Eq. 3) are unknown for glasses displaying heterogeneous nucleation, we cannot estimate the values of the surface energy for these two glasses.

However, other oxide glasses also have similar high values of density mismatch and still crystallise, although they always do with the crystal growth starting on heterogeneous nucleation sites that exist on the sample surfaces^54^. Hence, this large density mismatch is only an indication of the structural dissimilarity. One should then dive deeper into the structural details of the parent glass and crystal phases for further insight, as we will indeed do below.

### Structures of Albite Glass and Crystal

Ordering at short-length and, in some cases, at intermediate-length scales is a universal feature of the glassy state. Taylor and Brown^55^ indicated that the structure of Albite glass consists predominantly of 6-membered Si–O rings, whereas the structure of the isochemical crystalline compound is exclusively constructed of 4-membered rings.

According to Zanotto *et al*.^54^ the dipolar second moments, ΔM_2_/M_2C_, describing the spatial correlation between the network modifiers in oxide glasses and their isochemical crystals vary from approximately 4 to 16% for oxide systems that undergo internal homogenous crystal nucleation, whereas for those which only display surface nucleation, this parameter may reach 60%. However, for Albite glass and crystals, the Na-Na second moment differs by more than 130%! This is by far the largest difference ever reported for the modifier-modifier second moment among all oxide glass formers and clearly points to significant structural differences between the glass and crystal phases.

These results further support our working hypothesis that the intermediate and medium range structure of Albite glass and its corresponding crystal phase widely differ. However, this significant structural difference seems to hinder crystal nucleation only in said composition, not crystal growth, because Uhlmann *et al*.^10^, Selvaraj *et al*.^11^, and Liu *et al*.^12^ were able to crystallise Albite glass at high temperatures using crystalline seeds. This insensitiveness of crystal growth to the structural mismatch results from growth being normally measured at much higher temperatures (T ∼ T_m_) where bond breaking and rearrangement is much easier than at deep supercoolings, T ∼ T_g_, where the nucleation rates are normally measured.

### Structures of B_2_O_3_ Glass and Crystal

High-resolution nuclear magnetic resonance (NMR) measurements of Oxygen-17 in B_2_O_3_ glass^56^ provided evidence for structural units responsible for ordering on short- and intermediate-length scales. At the molecular level, planar BO_3/2_ units accounted for the local ordering. Oxygen-17 NMR spectra resolved detailed features of the inclusion of these units in boroxol rings, oxygen bridging two rings, and oxygen shared between two nonring BO_3/2_ units. On the basis of these and corroborative boron-11 NMR and scattering results, the authors concluded that boron oxide glass consists of domains that are rich or poor in boroxol rings. Approximately 70% of the boron atoms are located in boroxol-rich regions. The radius of the ring-rich domains was estimated to be about 20 Å or larger, which is quite similar to the sizes proposed from independent light scattering experiments and the Boson peak observed in Raman spectra. These domains were proposed to be the structural basis of intermediate-range order in glassy boron oxide.

A not yet well understood question is the relationship between the glassy and the various possible crystalline forms a system may adopt. By means of *ab initio* calculations, Ferlat *et al*.^57^ discovered the existence of B_2_O_3_ crystalline polymorphs with structural properties similar to the glass and formation energies comparable to the known ambient crystal. According to those authors, the energy degeneracy of the crystals, which is high at ambient pressure and suppressed under pressure, provides a framework to understand the system’s ability to vitrify and the origin of the crystallisation anomaly. Their main contribution was to evidence these novel crystals, some of them sharing greater structural similarity with the glass than do the experimentally synthesized crystals (B_2_O_3_-I). The ease of vitrification of B_2_O_3_ was related to the competition for crystallisation between numerous low-energy crystals (some with boroxols, some without). The authors considered that their work reaffirms the role played by polymorphism in a system’s ability to vitrify^58^,^59^.

From an experimental perspective, 80 years ago Kracek *et al*.^60^ prepared a B_2_O_3_ glass sample sprinkled with B_2_O_3_-I crystal seeds (this phase is not explicitly stated in their paper, but this is in agreement with its reported melting point of 450 °C). After several months at various temperatures, the crystals remained suspended and did not grow. As far as we know, no one has ever tried to seed B_2_O_3_ glass with B_2_O_3_-II crystals, but then the density mismatch would be even higher (3.11 g/cm^3^, giving almost 70% difference) or other polymorphs.

One important remark is that the structure of B_2_O_3_-I is exclusively made by BO_3_ triangles^61^, which is significantly different from the  ∼ 70% boroxol rings that the glass is made. In a recent publication, Wright and Vedisheva^62^ suggested that the excellent glass-forming ability of B_2_O_3_ is due to the need to either break up the boroxol groups to form crystalline B_2_O_3_-I, which has a structure based on ribbons of independent BO3 triangles, or to break up and reform boroxol groups to yield a suggested, but so far undiscovered, ambient pressure crystalline polymorph with a structure based solely on boroxols^62^.

This finding for the inorganic oxide glass-formers Albite and B_2_O_3_ is analogous to what is known by the polymer science community. A few high molecular weight organic materials, in particular atactic polymers (e.g., polystyrene and PMMA) are very resistant to crystallisation because their intricate chain entanglements prevent the required molecular reorientation to organize themselves into a crystalline lattice.

### Summary of Properties and Structural Aspects

Table 3 summarizes the main parameters that control the crystal nucleation rates. They are quite unfavourable for the two oxide glasses vindicating our hypothesis to explain the lack of observable crystallisation in these glasses.

Finally, it is quite interesting to note that Albite liquid is *strong*; its fragility index *m* is 26. However, other strong liquids, such as SiO_2_ and GeO_2_ easily crystallise when heated above T_g_. On the other hand, B_2_O_3_ can be considered a fragile glass-former (*m* ∼ 50) and is also notoriously reluctant to crystallise, whereas many other fragile liquids easily crystallise. Hence, the abnormal crystal nucleation behaviour is not linked to the liquid fragility.

## Conclusions

Our analysis clearly demonstrates that the lack of detectable crystallisation (“crystallisation anomaly”) in both supercooled liquids is indeed meagre homogeneous crystal nucleation, solving an enigma that persisted for 80 decades since the pioneering work of Kracek. This scenario is linked to the relatively small supercoolings where the glass transition takes place (T_g_/T_m_ = 0.76–0.78) in these compositions. The latter leads to a very small crystallisation driving force at T ≥ T_g_, where crystallisation is normally observable in laboratory timescales. Thus, a deeper supercooling is necessary to achieve a high enough crystallisation driving force, leading to a T_max_ that is well below the respective T_g_. Associated with this, we demonstrated that the crystal nucleation time-lags are extremely long at T_max_, effectively prohibiting that such experiments be conducted in laboratory time scales. However, for heterogeneous crystal nucleation catalysed by efficient nucleating agents, T_max_ is shifted to higher temperatures above the respective T_g_. In this setting, the predicted nucleation time-lags become measurable. These calculations explain the experimental results confirming that these glasses can be crystallised only when seeded with powerful nucleating agents. In the unseeded and ambient pressure conditions, they will only crystallise in geological time scales.

Moreover, this anomalous crystallisation behaviour is not linked with the liquid fragility, as both strong (Albite) and fragile (B_2_O_3_) liquids demonstrate this phenomenon. Ultimately, the major differences in the atomic structures of the parent liquids and their isochemical crystal phases are the cause for poor nucleation.

From a broader scientific and technological perspective, the present results not only debunk a long-standing mystery regarding the crystallisation anomaly, but also provide further insight into the connection between the structure and crystal nucleation ability of two important oxide glass forming liquids. In addition, these results could be used by glass researchers and engineers for the compositional design of oxide glasses that are stable against crystallisation. To that end, oxide glass-forming compositions should be selected that have a large Δρ/ρ_glass_ and large T_g_/T_m_.

## Additional Information

**How to cite this article:** Zanotto, E. D. and Cassar, D. R. The microscopic origin of the extreme glass-forming ability of Albite and B_2_O_3_. *Sci. Rep.* **7**, 43022; doi: 10.1038/srep43022 (2017).

**Publisher's note:** Springer Nature remains neutral with regard to jurisdictional claims in published maps and institutional affiliations.

## Figures and Tables

**Table 1:** Fitted values of the viscosity parameters for both liquids.

	η_∞,VFT_ (Pa.s.)	C_VFT_ (K)	T_0_ (K)	η_∞,CWAM_ (Pa s)	A (K)	B	T_g_ (K)
Albite	10^−5.44^	12,700	356	−3.25	9,302	1.66	1,085
B_2_O_3_	10^−0.97^	1,580	433	0.55	1,270	3.90	551

T_g_ is defined here as the temperature where the viscosity is equal to 10^12^ Pa.s.

**Table 2:** Some properties of glassy and crystalline Albite and B_2_O_3_.

	Albite	Source	B_2_O_3_	Source	unit
T_g_	1086	viscosity	554	viscosity	K
V_m_	100.3	106	27.3	107	cm^3^/mol
d_0_	5.5	calculated	3.6	calculated	Å
T_m_	1391	73	723	47	K
α	0.5	assumption	0.5	assumption	non-dimensional
ΔH_m_	59,300	108	24,070	47	J/mol
σ_cm_	0.16	calculated	0.16	calculated	J/m^2^
C_p,l_	369	75	128–0.0003*T*	46	J/K.mol
C_p,c_	Eq. (16)	46	Eq. (17)	47	J/K.mol

**Table 3:** Physical parameters controlling crystal nucleation in oxide glass formers that undergo homogeneous nucleation in comparison to Albite, B_2_O_3_, and LS2.

Parameter	Homogeneous nucleation	Albite	B_2_O_3_	LS2
T_gr_	<0.60	0.78	0.76	0.56
Viscosity at T_max,homo_ (θ = π rad) [Pa.s.]	≤10^12^ Pa.s.	∼10^24^	∼10^23^	∼10^12^
Viscosity at T_max,hetero_ (θ = π/7 rad) [Pa.s.]	<10^12^ Pa.s.	∼10^10^	∼10^7^	∼10^3^
ΔG(T_g_)/ΔH_m_	>0.4	<0.2	<0.2	0.42
Δρ/ρ_glass_ [%]	<8	15	34	4.3
